# ECM-Induced IL-23 Drives Immune Suppression in Breast Cancer via Regulating PD-1 on Tregs

**DOI:** 10.1186/s13046-025-03518-0

**Published:** 2025-09-01

**Authors:** Giovanna Talarico, Mara Lecchi, Anna Zanichelli, Paola Portararo, Laura Botti, Vera Cappelletti, Massimo Costanza, Annamaria Piva, Pietro Pratesi, Francesco Bertolini, Massimo Di Nicola, Claudio Tripodo, Valeria Cancila, Serenella Maria Pupa, Mario Paolo Colombo, Claudia Chiodoni, Paolo Verderio, Sabina Sangaletti

**Affiliations:** 1 Molecular Immunology Unit, Experimental Oncology Department, Fondazione IRCCS Istituto Nazionale Dei Tumori di Milano, Milan, Italy; 2 Bioinformatics and Biostatistics Unit, Department of Epidemiology and Data Science, Fondazione IRCCS Istituto Nazionale Dei Tumori Di Milano, Milan, Italy; 3 Neuroimmunology and Neuromuscular Diseases Unit, Fondazione IRCCS Istituto Neurologico Carlo Besta, Milan, Italy; 4 Laboratory of Hematology-Oncology, European Institute of Oncology IRCCS, Milan, Italy; 5 Medical Oncology Department, Fondazione IRCCS Istituto Nazionale Dei Tumori di Milano, Milan, Italy; 6 Tumor Immunology Unit, Department of Health Sciences, University of Palermo, Palermo, Italy; 7 Laboratory of Hematology-Oncology, European Institute of Oncology IRCCS, Milan, Italy; 8 Department of Experimental Oncology, European Institute of Oncology, Milan, Italy

**Keywords:** Breast cancer, Extracellular matrix, ECM3 signature, Tregs, IL-23, SPARC, Immune suppression, T cells, PD-1

## Abstract

**Background:**

High-grade breast cancer (HGBC) is an aggressive disease with poor prognosis, underscoring the need for new treatment strategies. The tumor microenvironment (TME), particularly the extracellular matrix (ECM), plays a pivotal role in tumor progression, therapy resistance, and immune regulation. An ECM-related gene signature (defined ECM3), found in approximately 35% of HGBC cases, is associated with aggressive tumors, epithelial-to-mesenchymal transition (EMT), poor clinical outcome and increased infiltration of immunosuppressive myeloid-derived suppressor cells (MDSCs).

**Methods:**

In this study, we investigated the impact of the ECM on T cell regulation in HGBC patients, focusing on the relationship between ECM3 + tumors and T cell phenotypes. We employed mouse models to dissect the molecular mechanisms linking ECM components to T cell regulation, with particular attention to the role of the matricellular protein SPARC, a key component of the ECM3 signature.

**Results:**

We revealed a significant correlation between highly suppressive programmed cell death-1 (PD-1) negative regulatory T cells (Tregs) and ECM3 + tumors. In mouse models, SPARC was found to down-regulate PD-1 on Tregs by promoting IL-23 release, which in turn induced SATB1 expression, a repressor of the *pdcd1* gene. The selective expression of the IL-23 receptor on Tregs accounted for the targeted effect of IL-23 on these cells. Notably, blocking IL-23 with monoclonal antibodies restored PD-1 expression on Tregs and activated T effector cells.

**Conclusion:**

These findings extend the immune-regulatory role of the ECM to include regulatory T cells and identify potential new therapeutic targets for high-grade breast cancers. Moreover, they highlight ECM3 as a potential biomarker of resistance to PD-1/PD-L1 immune checkpoint blockade (ICB), suggesting that ECM3⁺ patients may benefit from alternative checkpoint inhibitor therapies beyond PD-1/PD-L1.

**Graphical Abstract:**

A qPCR analysis of 8 genes was used to determine the ECM3 status in HGBC patients. The immunoprofile of circulating PBMCs revealed an enrichment of highly suppressive PD-1⁻ regulatory T cells in ECM3⁺ patients. Using murine models, we elucidated the mechanism linking ECM3 to PD-1⁻ Tregs: SPARC, a gene within the ECM3 signature, induces IL-23 in the tumor microenvironment. Through its cognate receptor, IL-23 promotes the transcription factor SATB1 in Tregs, which mediates the repression of PD-1.

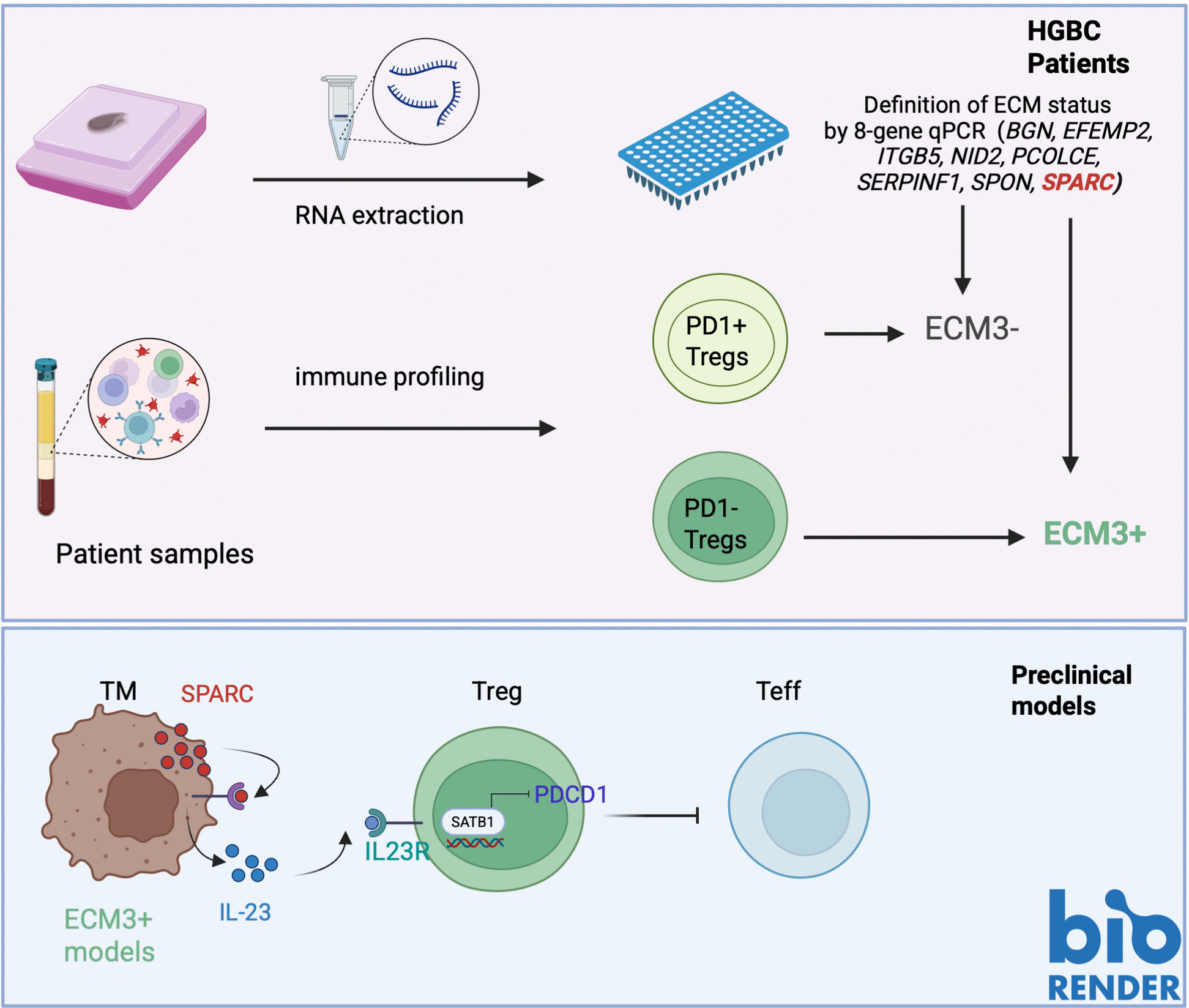

**Supplementary Information:**

The online version contains supplementary material available at 10.1186/s13046-025-03518-0.

## Background

The tumor microenvironment (TME) actively contributes to tumor initiation, progression, and resistance to current therapies. Among the noncellular components within the TME, the extracellular matrix (ECM) plays a pivotal role in various aspects of tumor progression, exerting a significant regulatory influence on tumor cells and the surrounding microenvironment. Specifically, the ECM has the capacity to impact all cancer hallmarks, including tumor growth, proliferation, metabolism, and immune surveillance [1]. Moreover, the composition of the ECM in breast cancer serves as a prognostic indicator, enabling the classification of patient subgroups with distinct clinical outcomes on the basis of the presence of specific ECM genes [2, 3].

Among the various ECM-related patterns, the ECM3 signature distinguishes aggressive tumors that exhibit characteristics of epithelial‒mesenchymal transition [4]. Importantly, the prognostic significance of the ECM3 signature remains unaffected by the molecular subtype of breast cancer, and it is linked to T-cell exclusion and increased infiltration of myeloid-derived suppressor cells (MDSCs) [5].

Overexpressing *SPARC*, a key gene within the ECM3 signature, enabled the modeling of ECM3 tumors in mice. This approach has revealed an increased abundance and altered spatial distribution of myeloid cells that, under the influence of SPARC, promote EMT and more aggressive histological phenotypes [6]. To date, extensive research has investigated the impact of SPARC on myeloid cell behavior, including features such as neutrophil activation and extracellular trap extrusion. However, the effects of SPARC and the ECM on T-cell function are poorly understood. The ECM has been shown to directly hinder T-cell migration, proliferation, and activation. This hindrance can occur through the ligation of LAIR receptors by type I collagen [7] or by the suppression of IL-2 production, with greater IL-2 release in softer substrates than in stiffer ones [8]. Interestingly, regulatory T cells (Tregs) use heparanase to access IL-2 sequestered by the ECM component heparan sulfate (HS) and exert immune suppression [9]. Nonetheless, the potential influence of the ECM on the function of adaptive T cells, particularly Tregs, remains poorly characterized.

In this study, we prospectively analyzed the peripheral blood (PB) of high-grade breast cancer (HGBC) patients enrolled in a prospective clinical study to determine whether the ECM3 classification correlates with Treg composition and activity. Additionally, using murine models of ECM3 HGBC, we investigated the mechanisms through which the ECM directly affects Treg function.

## Methods

### Sex as a biological variable

Our study focused exclusively on female patients/mice, as breast cancer predominantly affects females.

### Breast cancer patient samples

Human samples were collected in accordance with the Declaration of Helsinki, and the study was approved by the Ethics Committee for Clinical Research of the Fondazione IRCCS Istituto Nazionale dei Tumori (Approval number 167/17). All participants provided written informed consent before their inclusion in the study. Peripheral blood was obtained from consecutive HGBC patients (51 patients) and collected one day before surgery (Table 1).

**Table 1:** Clinical information of the patients enrolled in the study (Auth. Number 167/17). ER, estrogen receptor; PR, progesterone receptor; HER2, human epidermal growth factor receptor 2; NA, not available

	**N**	**%**
**Grading**		
II	11	21.57
III	39	76.47
unknown	1	1.96
**ER**		
+	29	56.86
-	19	37.25
unknown	3	5.88
**PGR**		
+	23	45.1
-	24	47.06
unknown	4	7.84
**Her2**		
0	10	19.61
1 +	15	29.41
2 +	9	17.65
3 +	14	27.45
unknown	3	5.88
**Her2 Fish**		
+	15	29.41
-	31	60.78
unknown	5	9.8
**Ki 67**		
+	43	84.31
-	5	9.8
unknown	3	5.88
**Subtype**		
HER 2 +	6	11.76
Luminal A	5	9.8
Luminal B	15	29.41
Luminal HER2	8	15.69
Triple negative	11	21.57
Insitu	3	5.88
unknown	3	5.88
**cT stage**		
T1	30	58.82
T2	14	27.45
T4	1	1.96
Tis	3	5.88
Tx	3	5.88
**cN stage**		
N0	34	66.67
N1	6	11.76
N2	1	1.96
Nx	10	19.61
**ECM3 status**		
+	21	41.18
-	30	58.82

### PBMC flow cytometry and cell sorting

Blood samples (6 ml) were collected in heparin tubes, and peripheral blood mononuclear cells (PBMCs) were obtained by diluting whole blood samples 1:2 with 1X PBS and subsequently subjected to density gradient stratification. The diluted whole blood samples were carefully layered onto Histopaque-1077 Ficoll (Sigma‒Aldrich) and centrifuged at 1,800 rpm for 30 min at room temperature (RT) without braking. Finally, the lymphocyte-enriched ring at the interface was transferred into a new collection tube and washed with 1X PBS by centrifugation at 1,200 rpm for 5 min at RT. PBMCs were then stained with a cocktail of antibodies to characterize different immune cell populations and analyzed with a Becton Dickinson Biosciences Celesta instrument (Supplementary Table 1).

### Cell lines, animals, and in vivo experiments

BALB/cAnNCrl mice (BALB/c) were purchased from Charles River Laboratories (Calco). All animal experiments were approved by the Institutional Ethics Committee for Animal Use (OPBA) and by the Italian Ministry of Health (authorization numbers 288/2017-PR) and were performed following to the 3Rs’ recommendations (Reduction, Refinement and Replacement). All experimental procedures were conducted in accordance with ethical guidelines and approved by the appropriate institutional review board. Measures were taken to minimize pain, suffering, and distress, including the use of anesthesia and analgesia where applicable, as well as the implementation of humane endpoints and routine monitoring for signs of discomfort. Animals were monitored for clinical signs including weight loss exceeding 20%, lethargy, abnormal posture or gait, decreased food or water intake, and signs of infection or inflammation. Mice were housed under pathogen-free conditions in the animal facilities at IRCCS Istituto Nazionale dei Tumori (Milan, Italy). Studies were performed on 6–8 week-old BALB/c immunocompetent female mice. A maximum number of 5 mice were housed in individually ventilated cages at a temperature of 23 °C, 40% humidity, with a circadian cycle of 12 h light/12 h dark. Water and food were provided ad libitum.

The mammary carcinoma cell line SN25A was obtained from *SPARC*-deficient mice that spontaneously developed mammary tumors due to the expression of the rat HER2/neu oncogene (BALB/c; SPARC < tm1Hwe > Tg(MMTV-Erbb2)NK1Mul/J). The cell lines were infected with the retroviral vector LXSPARCSH to overexpress *SPARC,* and the coisogenic cell line SN25ASP was obtained. The mice were injected into the mammary fat pad with SN25A (all at a dose of 2 × 10^5^ cells) and SN25ASP (10^6^ cells) cell lines. Another BC cell line, 4T1cl5 (SPARC-high), was obtained by subcloning 4T1 cells; its SPARC-negative counterpart was obtained through lentiviral delivery of *SPARC* shRNA (4T1cl5SP548). Primary tumor growth was measured twice a week with a caliper, and the volume was calculated via the formula d2XD/2, where d and D represent the short and long diameters, respectively. Tumors were collected when they reached a 10 mm diameter.

All the breast cancer cells were cultured in DMEM (Dulbecco's modified Eagle's medium; Thermo Fisher Scientific) supplemented with 10% fetal bovine serum (FBS; Thermo Fisher Scientific), 1% antibiotics (Thermo Fisher Scientific), 2 mM glutamine, 1 mM sodium pyruvate, 1 mM HEPES and 1 × minimum essential medium (MEM) without essential amino acid solution in a humidified atmosphere containing 5% CO2 at 37 °C.

### Western blot

The cells were trypsinized, collected in a Falcon tube, and centrifuged at 1500 rpm for 5 min at RT. The cells were lysed in RIPA buffer containing 50 mM Tris, pH 7.4, 1% NP-40, 150 mM NaCl, 1 mM EDTA, 1 mM Na3VO4 and protease inhibitor cocktail (Roche). Proteins were separated by SDS‒PAGE and subjected to immunoblotting. The cell lysates were microcentrifuged at 14,000 rpm for 20 min at 4 °C, and the supernatants were collected and stored at − 20 °C. For protein quantitation, the Bradford assay was used (Bradford, 1976). Five microliters of lysate or bovine serum albumin (BSA) was diluted in 195 μl of Bradford reagent and incubated for 5 min at room temperature. Serial dilutions of BSA were used as standard proteins in a 96-well plate. After incubation, the protein concentration was estimated from the absorbance at 595 nm. The protein concentration was determined via interpolation with the curve obtained with the standard BSA.

Thirty micrograms of total protein was separated on 4‒12 Bis‒Tris NuPAGE gels (Thermo Fisher Scientific). The cell lysates were mixed with 4x reducing SDS-Sample buffer and heated for 10 min at 99 °C. Before loading, protein samples were prepared by adding ZAP solution to 20–50 μg of protein, and the samples were denatured for 10 min at 99 °C. As a molecular weight standard, Page Ruler Plus prestained protein ladder (EuroClone) was used. Electrophoretic separation was achieved by applying a constant voltage in MOPS buffer. As suggested by the manufacturer, 500 μl of NuPAGE antioxidant was added to the chamber to protect the reduced disulfide bonds and sensitive amino acids from oxidation, thus allowing proper protein migration under reducing conditions. Proteins within the gels were then transferred onto nitrocellulose membranes (Amersham, Biosciences). Following blocking with 5% bovine serum albumin (BSA) and 0.1% Tween-20, the membranes were incubated overnight at 4 °C with the following antibodies: goat anti-mouse SPARC IgG (catalog #AF942, R&D Systems; dilution 1:500) and monoclonal mouse anti-mouse/human vinculin (clone hVIN-1, catalog #V9131, Sigma‒Aldrich; dilution 1:5000). After being rinsed with 0.1% Tween-20 in Tris-buffered saline (TBS), the membranes were incubated with the appropriate horseradish peroxidase-conjugated secondary antibodies: rabbit anti-goat IgG (catalog #R21459, Invitrogen; dilution 1:2000) and sheep anti-mouse IgG (catalog #NA931, Ge Healthcare; dilution 1:2000) for 1 h at RT. The membranes were incubated for 1 min with ECL Plus Western Blotting Substrate (Thermo Scientific) and then developed from X-ray film.

### Flow Cytometry Analysis

For FACS analysis, primary tumors (TM) and spleens (SPL) were collected and maintained in DMEM − 10% FBS and then minced and filtered through a 40 μm TM and 70 μm-pore cell strainer for SPL (BD). Red blood cells were removed via ammonium-chloride-potassium (ACK) lysing buffer containing 150 mM NH4Cl, 10 mM KHCO3, and 0.1 mM EDTA, which was subsequently resuspended in distilled H2O and filtered before use. The cells were stained with different anti-mouse monoclonal antibodies (Supplementary Table 2).

For intracellular staining, the Foxp3/Transcription Factor Staining Buffer Kit (Tonbo Biosciences) was used. Briefly, after surface marker staining, the cells were washed with FACS buffer (PBS 1X, 0.5% EDTA, 3% FBS), fixed and permeabilized for 30 min using the Foxp3/Transcription Factor Fix/Perm buffer provided with the kit. The samples were analyzed with a Becton Dickinson Biosciences LSR II Fortessa instrument. Flow cytometry data analyses were performed via FlowJo software (v10.2).

### Treg cell isolation via magnetic cell sorting

CD4 + CD25 + regulatory T cells were isolated from single-cell suspensions of SPLs, lymph nodes (LNs) and TMs from mice injected with SPARC-low/null (4T1 or 4T1cl5sp548) or SPARC-high (4T1SPARC and 4T1cl5) cells. The spleen, lymph nodes and tumors were disrupted with the plunger of a 1 ml syringe in a Petri dish filled with 2 ml of DMEM and then passed through a cell strainer. The cell suspensions were lysed with ACK, and after centrifugation, they were resuspended in a MACS buffer solution containing PBS (pH 7.2), 0.5% BSA and 2 mM EDTA. Mouse Treg cells were isolated via a CD4 + CD25 + Regulatory T-Cell Isolation Kit (Miltenyi Biotec, #130–091-041). Isolation was performed via a two-step procedure. First, non-CD4 + T cells are indirectly magnetically labeled with a cocktail of biotin-conjugated antibodies (CD4 + CD25 +) as the primary labeling reagent and antibiotin monoclonal antibodies conjugated to MicroBeads and CD25-PE as the secondary labeling reagent. After 20 min of incubation at 4 °C and centrifugation at 1500 r/min for 10 min, we discarded the supernatant, resuspended it by adding MACS solution and prepared an LD separation column in a MACS separator device. The LD separation column was washed twice with 1 ml of MACS solution, and then the cell suspension was added to the separation column to obtain the cells flowing from the sorting column.

The magnetically labeled non-CD4 + T cells are retained in the column, while the unlabeled CD4 + T cells run through. In the second step, the CD25 + PE-labeled cells are magnetically labeled with anti-PE microbeads and isolated by positive selection from the preenriched CD4 + cell fraction by separation over an MS MACS column, which is placed in the magnetic field of a MACS separator. The magnetically labeled CD4 + CD25 + cells were retained in the column, while the unlabeled CD4 + CD25– cells were passed through and used for suppression assays as responder T cells. After removing the column from the magnetic field, the magnetically retained CD4 + CD25 + cells were eluted as the positively selected cell fraction. Finally, we checked the purity via flow cytometry with CD4-APC (clone GK1.5) and CD25-PE (clone PC61.5) antibodies.

### Quantitative polymerase chain reaction (qPCR)

Total RNA was extracted via the Quick RNA Micro Prep Kit (Zymo Research) and subsequently quantified via a NanoDrop 2000c Spectrophotometer (Thermo Scientific). One microgram of total RNA was reverse-transcribed into cDNA via the MultiScribe-Reverse Transcriptase Kit (Applied Biosystems). Quantitative PCR was performed via the following Taqman Probes: *Il-23a* (Mm00518984_m1), *Il-10* (Mm01288386_m1), *Satb1* (Mm01268937_m1), *Pdcd1* (Mm01285677_g1), *beta-actin* (Mm02619580_g1), *IL-23a* (Hs00900828_g1), *Actb1* (Hs01060665_g1), and the Taqman Universal PCR Master Mix (Applied Biosystems). qPCR was performed on a 7900HT Fast Real-Time PCR System (Applied Biosystems).

### Treg cell suppression assay

To assess the suppressive potential of PD-1 + or PD-1^*neg*^ Tregs, LNs and SPL were isolated from BALB/c mice injected with 4T1SPARC BC cells. Tissues were pooled, homogenized and sorted on the basis of CD4 + (APC, clone GK1.5), CD25 + (PE, clone PC61.5) and PD-1 (FITC, clone RMP1-30) surface expression into Treg PD-1 + and Treg PD-1 ^*neg*^ populations. CD4 effector T cells and Tregs from naïve mice were isolated through magnetic separation (Miltenyi Biotec, described in Materials and Methods Sect. 3.5). Teff and Treg cells (1:1 ratio) were isolated from BALB/c mice via stimulation with mitomycin C-treated pooled cells such as APCs and 1 µg/ml anti-CD3 mAb. The cells were cultured in round-bottom 96-well plates at a density of 4 × 10^5^ cells/well in 200 µl of RPMI 1640 (EuroClone) supplemented with L-glutamine (2 mM), sodium pyruvate (1 mM), nonessential amino acids (0.1 mM), penicillin (100 U/ml), streptomycin (0.1 mg/ml), 2-ME (5 × 105 M), HEPES buffer (0.01 M) and 10% FBS (enriched with RPMI 1640). After 72 h of incubation at 37 °C with 5% CO_2_, the cultures were pulsed for 9 h with 0.5 µCi of [3H]-thymidine per well, and proliferation was measured from triplicate harvest cultures with a commercial cell harvester and determined with an n-counter (PerkinElmer). The data are expressed as the mean count per minute (c.p.m.).

### Immunolocalization analyses

Four-micrometer–thick human tissue sections were deparaffinized, rehydrated, and unmasked using Novocastra Epitope Retrieval Solutions at pH9 (Leica Biosystems) in a thermostatic bath at 98 °C for 30 min. The sections were then brought to room temperature and washed in PBS. After neutralization of the endogenous peroxidase with 3% H2O2 and Fc blocking with 0.4% casein in PBS (Leica Biosystems), the sections were incubated with mouse monoclonal CD8 (clone 4B11, 1:50 pH9, catalog no. NCL-L-CD8-4B11, Leica Biosystems) primary antibody.

Immunohistochemistry (IHC) staining was developed using the Novolink Polymer Detection Systems (Leica Biosystems) and DAB (3,3′-Diaminobenzidine, Novocastra) as substrate chromogen. IHC-stained slides were analyzed and imaged under a Zeiss Axioscope-A1 and Axiocam 503 Color camera (Zeiss).

### Cytospin

SN25A, SN25ASPARC, 4T1, and 4T1SPARC cells were detached and suspended at 5 × 10^5^/200 μl. Glass slides were mounted with a paper pad and cuvettes with a metal holder, loaded with 200 μl of cell suspension and then spun for 3 min at 800 rpm with a cytocentrifuge. After the cuvettes and filters were removed, the slides were dried overnight and then fixed for 10 min with 4% PFA. For immunofluorescence, after permeabilization for 5 min with PBS containing 0.1% Triton X-100 (Sigma) and blocking with PBS containing 5% BSA, the samples were incubated with an anti-IL-23 antibody (Ab45420, Abcam) at a 1:250 dilution in PBS containing 2% BSA for 1 h at room temperature. An Alexa Fluor 488-conjugated goat anti-rabbit polyclonal IgG (Invitrogen, 1:500) was used as the secondary antibody for 45 min at room temperature. The slides were mounted with ProLong Diamond Antifade Mountant with DAPI (Thermo Fisher Scientific) and acquired with a Leica DM4 B microscope equipped with a Leica DFC450 C digital camera utilizing LAS X software (Leica Biosystems).

### Gene expression profile analysis

Gene expression profiling was performed via the Thermo Fisher Mouse Clariom S Assay. The raw data were preprocessed via the SST-RMA algorithm provided in the Transcriptome Analysis Console software (Thermo Fisher). Downstream analyses were conducted on the preprocessed data via R software. For genes represented by multiple probes, the probe with the highest variance across samples was selected via the collapseRows function from the WGCNA package [10].

To assess gene expression correlation, log2-transformed expression data were used, and correlations were computed via Pearson’s correlation.

For single-cell analysis, we utilized a publicly available dataset [11] deposited in the NCBI Gene Expression Omnibus (GEO) under the accession number GSE110686. The dataset comprises FACS-sorted CD3 + T cells from primary tumor tissues of two triple-negative breast cancer patients. The cell ranger output matrices were downloaded from GEO, and subsequent analyses were conducted via the Scanpy library in Python. Quality controls were performed following the same filtering steps described by the original authors for consistency. The data were then normalized by total counts and log transformed. The batch effect between samples was corrected via the combat function, and unwanted sources of variation (derived from total counts and mitochondrial gene percentage) were regressed out. Cell type annotation was derived from the single-cell RNA-seq database Tumor Immune Single-cell Hub 2 (TISCH2, link: http://tisch.comp-genomics.org/home/).

### Statistical analysis

For the analysis of the prospective cohort, the qPCR expression levels of the 8 genes included in the ECM3-reduced signature (33523584) together with the housekeeping gene RPLP1 were used to classify patients as ECM3 + and ECM3-.

The associations between the ECM3 status or SPARC level (high vs low, according to the median of the distribution) and each immune cell population were first explored via the nonparametric Wilcoxon test (W). Populations found to be relevant were subsequently investigated by applying a t test on the opportunely transformed values [12]. The same approach was used to evaluate the associations between ECM3 status and SATB1/PDCD1 gene expressions, in ECTO case series. All the statistical analyses were carried out with SAS® Studio software (Release 5.2.; SAS Institute, Inc., Cary, NC, USA) by considering a significance level of alpha = 0.05 via a two- or one-sided test, according to the underlying hypothesis.

For in vitro and ex vivo experiments, dot plots reporting means with standard deviations (SDs) or boxplots, according to the number of observations, were plotted to describe the data. Differences between groups of interest were assessed with nonparametric Wilcoxon (W) or Kruskal‒Wallis (KW) tests; p values were estimated through exact tests or the Monte Carlo approach to handle sample size limitations, if any.

During data analysis, no data points were excluded unless they resulted from technical issues, which were documented and predefined in the analysis plan.

## Results

### Higher presence of *Ki67 + PD-1 *^neg^ T regulatory cells defines *ECM3 + * patients

We have previously demonstrated that the ECM3 signature correlates with a localized immune suppressive microenvironment characterized by enrichment of myeloid-derived suppressor cells and exclusion of T cells [6]. In order to investigate whether variations in the extracellular matrix composition might influence the phenotype of circulating myeloid cells and T lymphocytes, we conducted a prospective study involving 51 consecutive breast cancer (BC) patients (Table 1).

Tumor biopsies were analyzed for ECM3 status using an 8-gene qPCR-based assay, as described previously [5]. Among these samples, 21 were classified as ECM3 +, whereas the remaining 30 were classified as ECM3-. All 51 patients underwent peripheral blood mononuclear cell (PBMC) analysis via multiparametric flow cytometry (FACS) to evaluate the contents of MDSCs, regulatory T cells (Tregs), and CD4 and CD8 T cells.

**Fig. 1:**
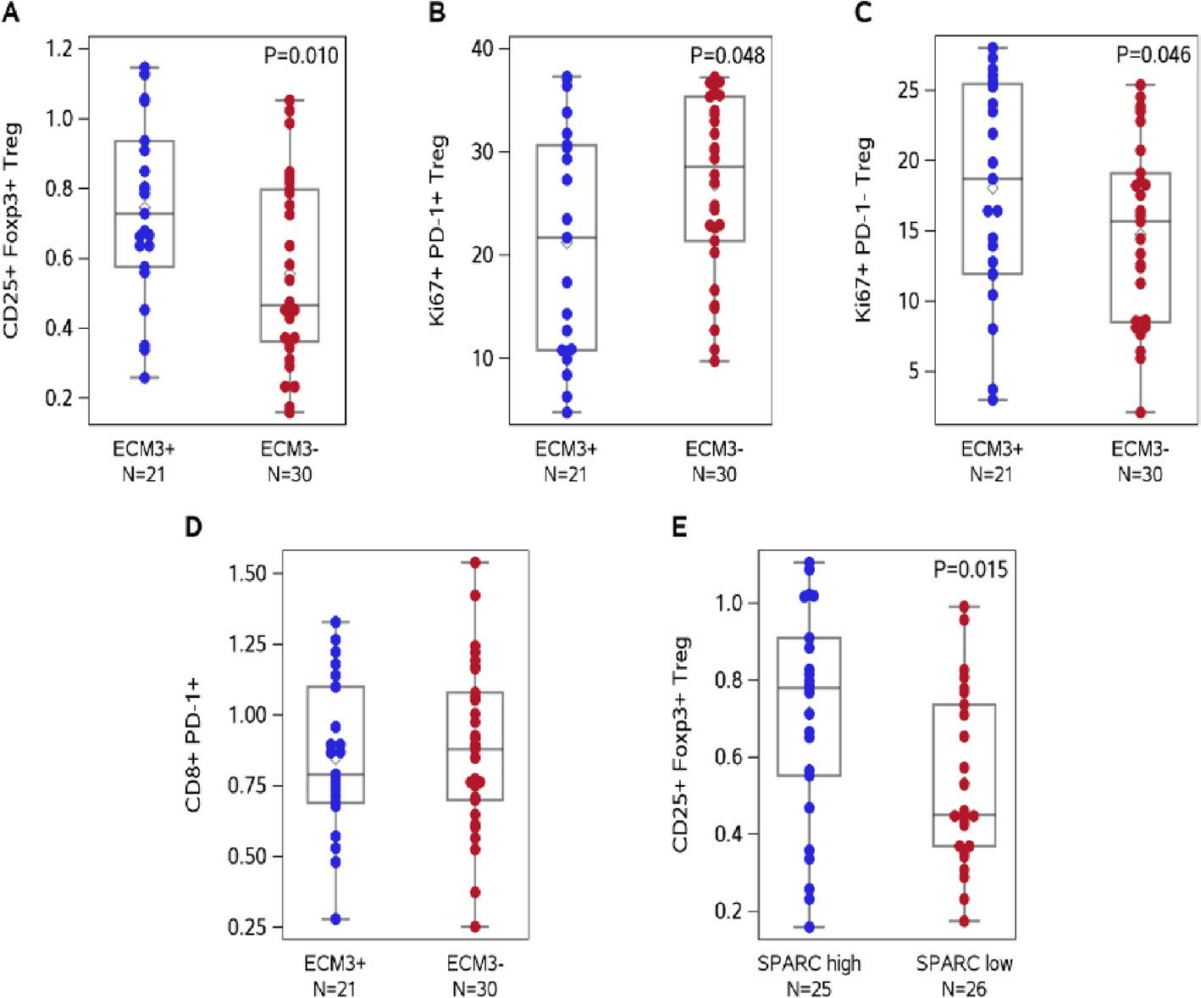
*Ki67* + *PD-1*^*neg*^* regulatory T cells are enriched in ECM3* + *patients.* Distributions of Box‒Cox-transformed immune cell populations according to the ECM3 status or SPARC level. Each box indicates the 25th and 75th percentiles. The horizontal line inside the box indicates the median, and the whiskers indicate the extreme values. Box‒Cox transformation parameters are equal to λ = −0.5 (**A**; *p*-value: 0.010), λ = 0.75 (**B**; *p*-value: 0.048), λ = 0.65 (**C**; *p*-value: 0.046), λ = −0.5 (**D**; *p*-value: 0.586), and λ = −0.5 (**E**; *p*-value: 0.015). *P*-values were estimated using Student’s t-test

Statistical analysis revealed that the distribution of CD25 + Foxp3 + Treg (p value 0.010) cells was significantly greater in the ECM3 + patients (Supplementary Fig. 1 for gating strategy). Additionally, we detected significant associations between ECM3 status and Treg Ki67 + PD-1 + cells (p value 0.048) and between Treg Ki67 + PD-1 ^*neg*^ (p value 0.046), which were under- or overrepresented in the ECM3 + group, respectively (Fig. 1A‒C).

Given the association between ECM3 status and PD-1 expression on Tregs, we also assessed exhausted PD-1 + CD8 + T cells in ECM3 + patients. The results indicated that the expression of PD-1 on CD8 + T cells was not significantly associated with ECM3 status (p value 0.586, Fig. 1D).

### SPARC is responsible for Treg *Ki6 + PD-1*^neg^ expansion in the ECM3 BC model

To investigate the mechanisms leading to *PD-1 *^*neg*^ Treg generation in ECM3 + tumors, we employed murine BC cell lines expressing different levels of the matricellular protein SPARC (secreted protein acidic and rich in cysteine, also known as osteonectin or BM-40), the leading gene defining the ECM3 phenotype [6]. Notably, the expression level of this gene is, per se, correlated with the frequency of circulating Tregs in ECM3 + patients, which is in line with its possible functional activity (p value 0.019; Fig. 1E).

**Fig. 2:**
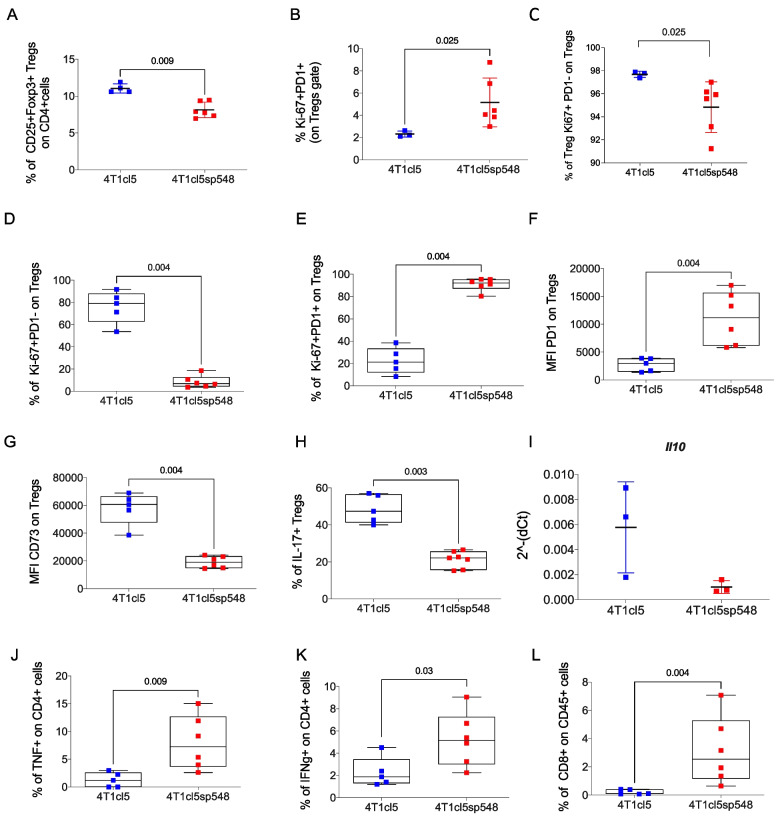
*Expansion of Ki67* + *PD-1 *^*neg*^* Tregs in 4T1cl5 and 4T1cl5sp548 BC models.* **A**. Frequency of Tregs in the PB of mice injected with 4T1cl5 and 4T1cl5sp548 cells. Tregs were defined as CD25 + Foxp3 + withing CD4 + T cell gate (p-value: 0.009); **B-C.** Frequency of Ki-67 + PD-1 + (p-value: 0.025) and of Ki-67 + *PD-1*^*neg*^ (*p*-value: 0.025) Tregs in PB. **D-E.** Frequency of Ki67 + PD-1 + and of Ki67 + *PD-1*.^*neg*^ Tregs(*p*-value: 0.004) in the TME. **F**. Mean fluorescence intensity (MFI) of PD-1 on Tregs in 4T1cl5 or 4T1cl5sp548 tumors (*p*-value: 0.004). **G**. MFI of CD73 on tumor-associated Tregs (*p*-value: 0.004). **H**. Frequency of IL-17 + Treg cells (*p*-value: 0.003). **I**. qPCR analysis of *Il10* on FACS-sorted and pooled Tregs (*p*-value: 0.099). **J-K.** Frequencies of CD4 + TNF + (p-value: 0.009) and of CD4 + IFNγ + T cells (*p*-value: 0.003); L. Frequency of total CD8 + T cells (*p*-value: 0.004) in ex vivo isolated 4T1cl5 and 4T1cl5sp548 tumors. *P*-values were estimated using nonparametric Wilcoxon test. (4T1cl5 n = 5; 4T1cl5sp548 n = 6)

We started our analysis using 4T1cl5 cells, a subclone of parental 4T1 cells, which display greater metastatic potential and, unlike the 4T1 line, elevated levels of SPARC [13]. As a nonmetastatic counterpart, we employed a SPARC-silenced shRNA variant of this subclone (4T1cl5sp548) (Supplementary Fig. 2 A). Immunocompetent syngeneic BALB/c mice were injected with either 4T1cl5sp548 or 4T1cl5 cells and sacrificed after 28 days for the analysis of PB and TME Treg cell distributions. PB analysis revealed an increased frequency of CD25 + Foxp3 + Tregs in mice bearing 4T1cl5 tumors, and, consistent with data from patients, we observed significant changes in the fraction of Ki67 + Tregs with a reduction in the frequency of the Ki67 + PD-1 + population and a concomitant increase in the frequency of Ki67 + PD-1^*neg*^ Tregs in these mice (Fig. 2A‒C).

We next evaluated the TME of 4T1cl5 and 4T1cl5sp548 tumors to determine whether these changes in circulating Treg populations also occur locally and to verify whether the enrichment of PD-1^*neg*^ Tregs was associated with a less or more suppressive TME. The results depicted in Fig. 2D confirmed that, compared with SPARC-silenced 4T1cl5sp548 tumors, 4T1cl5 tumors were enriched with Ki-67 + PD-1^*neg*^ cells. Conversely, the number of Ki67^+^ PD-1 + cells was significantly greater in 4T1cl5sp548 tumors (Fig. 2E). Consistent with these findings, the mean fluorescence intensity (MFI) of PD-1 on Tregs was reduced in SPARC-high tumors (Fig. 2F).

Given that the impact of PD-1 on Tregs is still debated, we evaluated the concomitant expression of CD73, a cell-surface ectoenzyme associated with Treg suppressive properties, and relevant cytokines, such as IL-10 and IL-17, which contribute to the identification of tolerogenic Tregs [14]. The results revealed that the Ki67^+^ PD-1^*neg*^ Treg population expressed high levels of CD73 (Fig. 2G) and *IL-17* (Fig. 2H), as well as a trend toward increased *IL-10* expression (Fig. 2I), the latter of which was detected through qPCR analysis of FACS-sorted and pooled Tregs. Overall, these data suggest that PD-1^*neg*^ Tregs may exert stronger suppressive effects than their PD-1 + counterparts do. Consistently, SPARC-high tumors presented fewer activated CD4 + T cells that produced TNF (Fig. 2J) and IFNg (Fig. 2K) and reduced infiltration of CD8 + T cells (Fig. 2L).

**Fig. 3:**
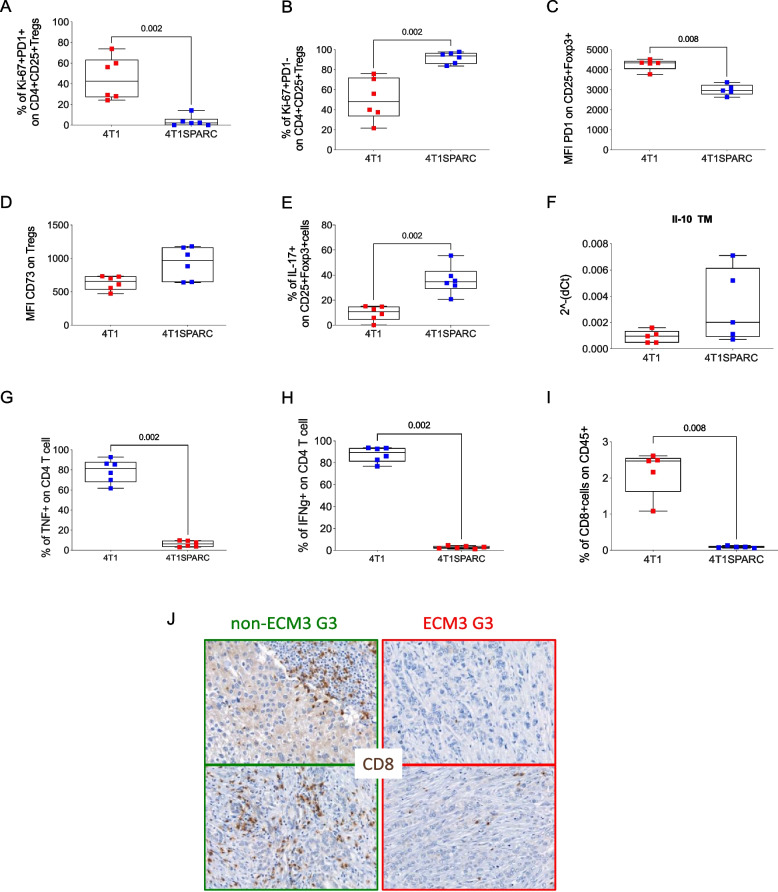
*Expansion of Ki67* + *PD-1 *^*neg*^* Tregs in 4T1and 4T1SPARC BC models* **A-B**. Frequencies of Ki-67 + PD-1 + (*p*-value: 0.002) and of Ki-67 + *PD-1*^*neg*^ Tregs in the PB of mice injected with 4T1 or 4T1SPARC cells (*p*-value: 0.002). **C-D**. MFI of PD-1 (*p*-value: 0.008) and of CD73 (*p*-value: 0.065) on CD25 + Foxp3 + Tregs in ex vivo isolated 4T1 or 4T1SPARC tumors. **E**. Frequency of Tregs producing IL-17(*p*-value: 0.002). **F**. qPCR analysis for *Il10* expression in FACS-sorted Tregs (*p*-value: 0.087). **G-I**. Frequencies of CD4 + TNF + (*p*-value: 0.002), CD4 + IFNγ + (*p*-value: 0.002), and of total CD8 + T cells (*p*-value: 0.008) in 4T1 or 4T1SPARC tumors. *P*-values were estimated using nonparametric Wilcoxon test. (*n* = 6/group). **J**. IHC analysis of CD8 + T cells in ECM3 + and ECM3- patient tumor biopsies

Supporting findings were obtained from an examination of mice injected with parental 4T1 cells, which express low levels of SPARC, and their SPARC-overexpressing counterpart (4T1SPARC), which was obtained through retroviral vector infection (Supplementary Fig. 2 A). Increased expansion of Ki-67 + PD-1^*neg*^ Tregs (Fig. 3A-B) along with a reduction in the MFI of PD-1 on Tregs (Fig. 3C) occurred in 4T1SPARC tumors. The MFI of CD73 on PD-1^*neg*^ Tregs (Fig. 3D, not statistically significant) and the production of IL-17 (Fig. 3E) and *IL-10* (Fig. 3F, not statistically significant) were also increased in these tumors. In the other model, this phenotype was associated with decreased activation of effector T cells, which release TNF-α or IFNγ (Fig. 3G-H). Furthermore, as in the 4T1cl5 tumors, the number of CD8 + T cells was lower in the 4T1SPARC tumors than in the 4T1 and SPARClow counterparts (Fig. 3I).

Notably, the data from murine models are in line with those from patients in which the representative IHC analysis performed on tumor biopsies from ECM3 + and ECM3- patients clearly revealed a reduction in CD8 + T cells in ECM3 + tumors (Fig. 3 J).

Finally, for further validation, we analyzed another pair of tumor cell lines derived from SPARC-null HER2Neu transgenic mice and their SPARC-transduced coisogenic variants, SN25A and SN25ASP [6]. The data in Supplementary Fig. 2B confirmed the direct effect of SPARC on PD-1 expression downregulation in Tregs.

### *PD-1 *^neg^ Treg cells are more suppressive than their PD-1* +* counterparts

**Fig. 4:**
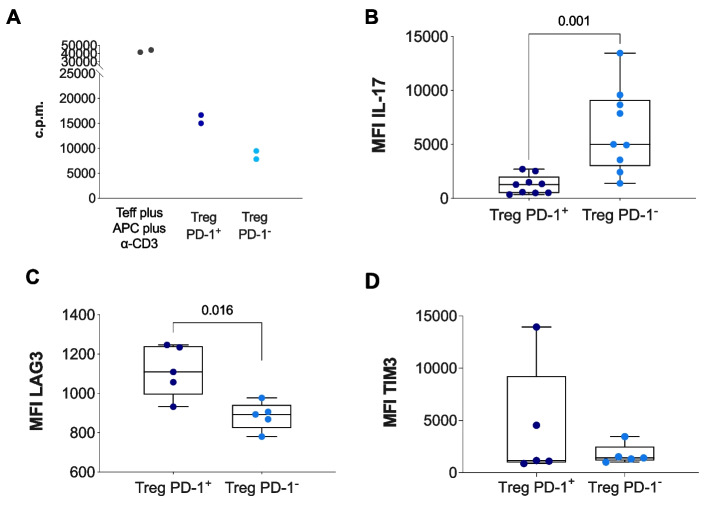
*PD-1*^*neg*^* Treg cells are more suppressive than their PD-1* + *counterparts.* **A.** Proliferation of T cells cultured in vitro with Treg cells isolated from SPARC-high (4T1SP) and SPARC-low (4T1) mice. T cells were stimulated with soluble anti-CD3 in the presence of splenocytes as antigen-presenting cells (APCs) and pulsed with 0.5 µM H3-thyminide per well for 18 h, and proliferation was measured in duplicate on a b-counter. C.p.m. = proliferation in counts per minute (no statistical test applied due to insufficient sample size; *n* = 2). **B**. MFI of IL-17 (*p*-value: 0.001), LAG3 (**C;** *p*-value: 0.016), and TIM3 (**D;** *p*-value: 1) on *PD-1*^*neg*^* or PD-1* + *Tregs* isolated from the LNs of 4T1SP-bearing mice. *P*-values were estimated using nonparametric Wilcoxon test

To functionally characterize PD-1^*neg*^ Treg cells, we evaluated their suppressive activity in vitro in comparison with that of their PD-1 + Treg counterparts. Treg cells were FACS-sorted from the lymph nodes (LNs) of mice injected with either SPARC-high (4T1SPARC) or SPARC-low (4T1) cells, checked for purity, and then cultured with naïve T effector (Teff) cells (CD4 + CD25-) in the presence of splenocytes, antigen-presenting cells (APCs), and soluble anti-CD3 for 72 h. The results revealed that PD-1^*neg*^ Treg cells were more efficient than their PD-1 + counterparts in suppressing Teff cell proliferation (Fig. 4A). In line with this suppressive phenotype, PD-1^*neg*^ Tregs isolated from the LNs of 4T1SPARC-bearing mice expressed significantly higher levels of IL-17 (Fig. 4B) and lower expression levels of the exhaustion markers LAG3 (Fig. 4C) than PD-1 + Tregs did, along with a trend toward TIM3 reduction (Fig. 4D).

### *PD-1 *^neg^Tregs display increased levels of SATB1

To gain insight into the mechanisms linking SPARC with the downmodulation of PD-1 on Tregs, we focused on SATB1, a nuclear epigenetic regulator relevant for T-cell development and function [15]. Notably, SATB1 has been shown to repress PD-1 expression in tumor-reactive T cells [16]. Therefore, we hypothesized that *SATB1* might also be responsible for PD-1 downregulation in Tregs from SPARC-high tumors.

**Fig. 5:**
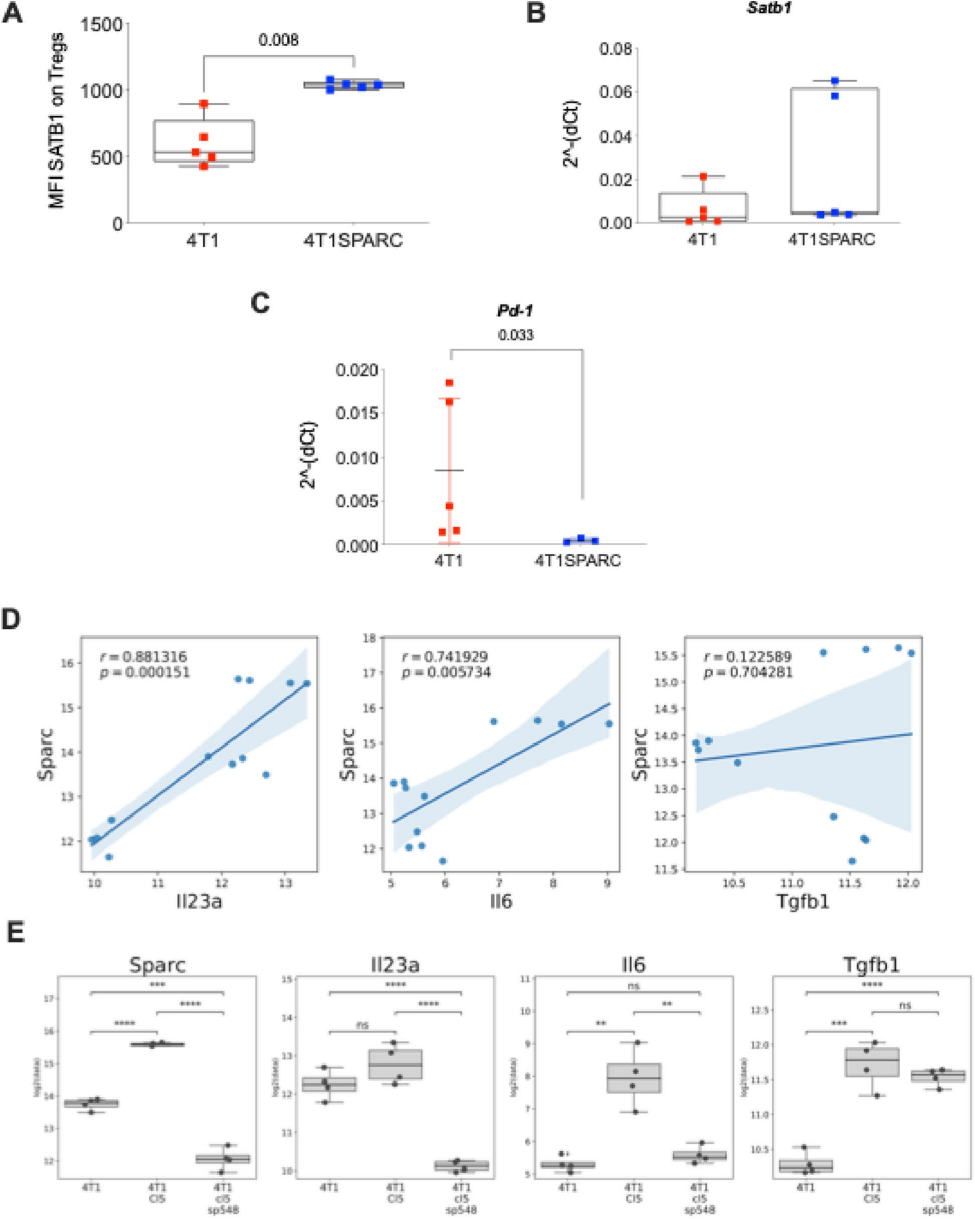
*PD-1*^*neg*^* Tregs display increased levels of SATB1.* **A***.* MFI of SATB1 in Tregs from SPARC-high tumors (4T1SP) compared with those isolated from their SPARC-low parental counterparts (4T1; *p*-value: 0.008). **B**. Semiquantitative qPCR analysis of *Satb1* expression in FACS-sorted intratumor Tregs isolated from 4T1SP and 4T1 mice (*p*-value: 0.222). **C.** Semiquantitative qPCR analysis of *Pd1* expression in FACS-sorted intratumor Tregs isolated from 4T1SP and 4T1 mice (p-value: 0.033). P-values were estimated using nonparametric Wilcoxon test. **D.** Correlation plot between *SPARC* expression (y-axis) and *Il-23a* (left), *Il6* (center) and *Tgfb1* (right) expression (x-axis) in the 4t1, 4t1cl5 and 4T1cl5sp548 samples. Each point represents an individual sample, the regression line depicts the linear correlation between the two genes, and the shadow area represents the 95% confidence interval of the regression fit. (statistic: Pearson correlation). **E**. Boxplot of gene expression among the three conditions (4t1, 4t1cl5 and 4T1cl5sp548 cells). Each boxplot displays the median and interquartile range; dots represent the individual data points. P-values were estimated using two-sided T-test (*: 1.00e-02 < *p* < = 5.00e-02; **: 1.00e-03 < *p* < = 1.00e-02; ***: 1.00e-04 < *p* < = 1.00e-03; ****: *p* < = 1.00e-04)

FACS analysis revealed an increased MFI of SATB1 in Tregs from 4T1SPARC tumors compared with SPARC-low parental 4T1 cells (Fig. 5A). Semiquantitative qPCR analysis of FACS-sorted intratumor Tregs confirmed a trend toward increased *Satb1* expression (Fig. 5B) in Tregs isolated from SPARC-high tumors (4T1SPARC) compared with those isolated from their SPARC-low counterparts (4T1). Notably, these Tregs also displayed decreased *Pd1* mRNA expression (Fig. 5C). The same results were obtained for 4T1cl5 cells and their *SPARC*-knockdown counterparts (Supplementary Fig. 2C-D).

To identify the mechanism involved in SPARC regulation of *Satb1* expression, we performed gene expression profile analysis of 4T1, 4T1cl5 and 4T1cl5sp548 cells characterized by different levels of SPARC expression. This analysis revealed a direct correlation between the expression of *Sparc* and *Il23*, which is a known key regulator of *Satb1* [17, 18]. We also examined TGFb1, another possible SATB1 regulator, without finding any correlation with the level of S*parc* expression. Furthermore, high expression of *Il-23* was detected in 4T1 and 4T1cl5 cells, whereas total downregulation of Il-23 was detected in their *sparc*-silenced counterparts. Additionally, the same trend was observed for *Il-23*, a gene whose expression was completely downregulated in *sparc-*silenced cells (Fig. 5 D-E).

### IL-23 mediates SPARC-regulated SATB1 expression in Tregs

**Fig. 6:**
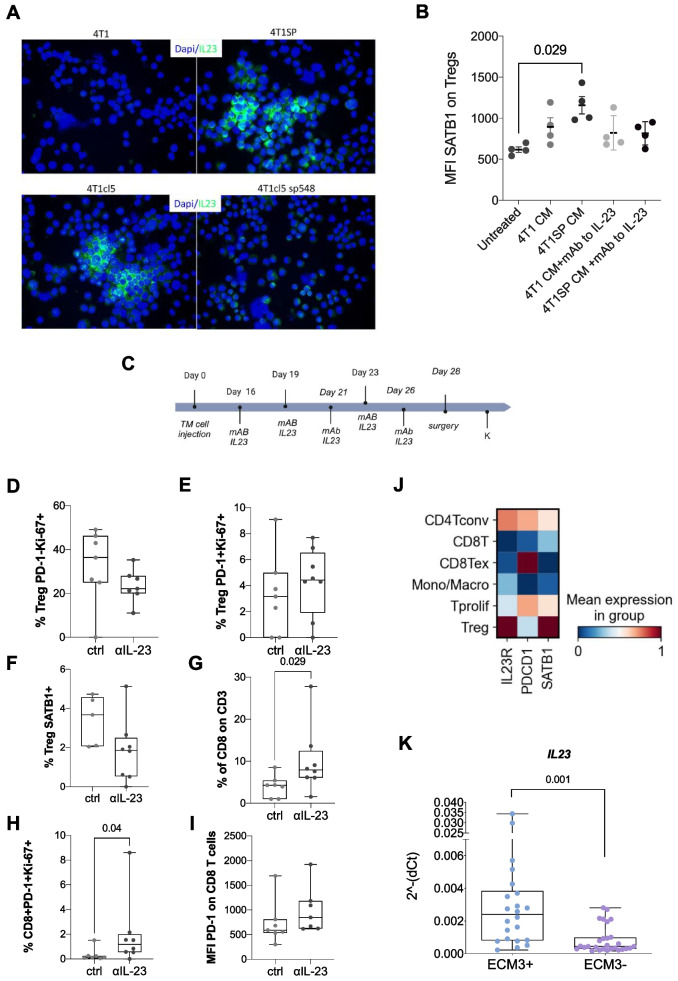
SPARC regulation of IL23 controls SATB1 expression in Tregs. **A***.* Immunofluorescence (IF) analysis for IL23 of 4T1, 4T1SP, 4T1cl5, and 41c15sp548 BC cells. **B.** MFI of SATB1 in naïve Tregs cocultured with TM supernatants from SPARC-high tumor (4T1SP) cells and their SPARC-low parental counterpart (4T1) alone or in combination with a monoclonal antibody blocking IL-23. The untreated samples were used as experimental controls (Kruskal‒Wallis test, p-value: 0.005; Wilcoxon test, p-value: 0.029). C. 28-day treatment scheme of B/c mice injected with 4T1cl5 cells and treated with anti-IL-23 monoclonal antibody in a neoadjuvant setting. D-F. FACS analysis showing the frequency of Ki-67 + PD-1neg Tregs (*p*-value: 0.165), Ki-67 + PD-1 + Tregs (*p*-value: 0.394) and of SATB1 + Tregs (*p*-value: 0.065) in the PB of mice treated with mAb to IL23 (*n* = 8) or the isotype control (*n* = 7). **G-H.** Frequency of total CD8 + cells (*p*-value: 0.029)and of Ki-67 + PD-1 + CD8 + T cells (*p*-value: 0.004); panel I shows the MFI of PD-1 on CD8 + T cells (*p*-value: 0.128). **J.** Heatmap illustrating the expression levels of the *IL-23R, PDCD1* and *SATB1* genes in breast cancer on the basis of a single-cell RNA-seq dataset (GSE110686). Each row shows the average gene expression after grouping the cells by cell type. Gene expression has been standardized between 0 and 1 across cell types (CD4Tconv = conventional CD4 + T cells; CD8Tex = exhausted CD8 + T cells; Tprolif = proliferating T cells; Mono/Macro: monocytes and macrophages). **K**. Semiquantitative qPCR analysis of *Il23* expression in ECM3 + and ECM3- patient tumor biopsies (*p*-value: 0.001). P-values were estimated using nonparametric Wilcoxon or Kruskal Wallis tests

The increased IL-23 expression in SPARC-high tumors was confirmed by qPCR and, at the protein level, by immunofluorescence (IF) (Fig. 6A and Supplementary Fig. 3A-B). In support of these findings, we found the same association between *SPARC* and *Il-23* in the third BC model represented by SN25A (*Sparc*-KO) and SN25ASP (*Sparc*-high) breast cancer cells [6] (Supplementary Fig. 3C-D). Mechanistically, the treatment of naïve Tregs with SPARC-high, but not SPARC-low, TM-supernatants increased the expression of *Satb1,* and the addition of a monoclonal antibody blocking IL-23 reversed this effect (Fig. 6B). Collectively, these results suggest that IL-23 functions as the key mediator of SPARC-regulated *Satb1* expression in Tregs.

Finally, to determine whether blocking IL-23 was sufficient to reverse immune suppression in vivo, B/c mice were injected with 4T1cl5 cells. Once tumors reached approximately 4 mm^3^ in size, mice were randomized and treated with anti-IL-23 antibodies in a neoadjuvant setting. Assignment to the groups was done blindly by the experimenters to minimize bias during treatment administration and outcome assessment (Fig. 6C).

Changes in the TME were evaluated after surgical removal of the primary tumor after 10 days of treatment with an anti-IL-23 antibody. A reduction in the frequency of suppressive PD-1^*neg*^ Ki67 + Tregs (Fig. 6D) paralled by a trend of increased frequency of less suppressive PD-1 + Ki67 + Tregs (Fig. 6E) and a non-significant reduction of SATB1 (Fig. 6F) characterized anti-IL-23 treated tumors. Confirming that IL-23 blockade results in a less suppressive TME engendered by Tregs, the frequencies of total CD8 + T cells (Fig. 6G) and activated PD-1 + Ki-67 + CD8 + T cells (Fig. 6H) were greater in these tumors. Notably, FACS analysis also revealed that anti-IL-23 treatment did not significantly affect PD-1 expression on CD8 + T cells (Fig. 6I). A possible explanation stems from GEP analysis of a scRNA-seq dataset (GSE110686), which revealed that CD8 T cells express *PDCD1* but not *IL-23R* or *SATB1*. In contrast, Tregs expressed *IL-23R and SATB1* but expressed lower levels of *PDCD1* (Fig. 6J).

To provide confirmatory data on the link between SPARC and IL-23 in patients, we evaluated *IL-23* mRNA levels via semiquantitative qPCR in tumor biopsies from the prospective BC cohort, which revealed significantly increased expression of *IL-23* in ECM3 + tumors (Fig. 6K).

**Fig. 7:**
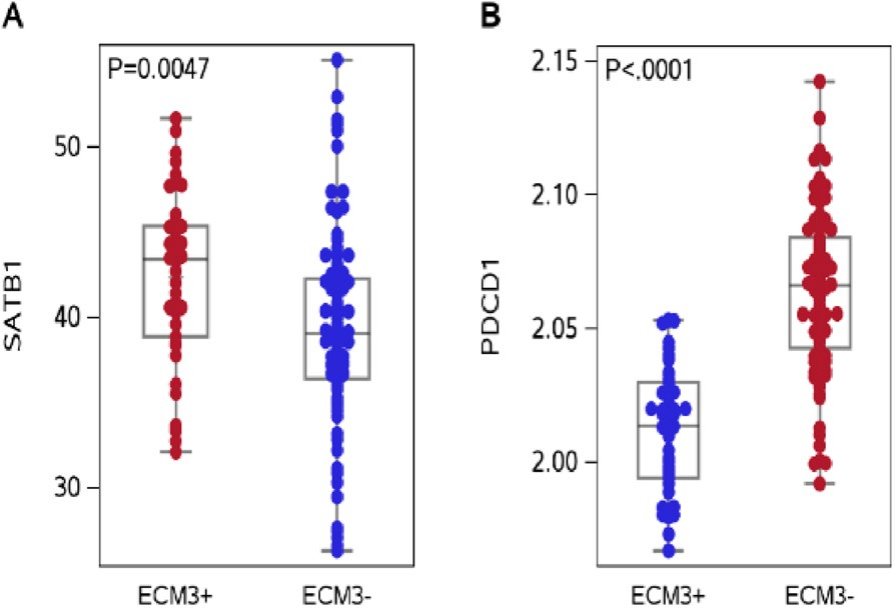
*Expression of PD1 and SATB1 in ECM3* + *and ECM3- HGBC dataset*. Distributions of Box‒*Cox*-transformed genes expressions according to the ECM3 status. Each box indicates the 25th and 75th percentiles. The horizontal line inside the box indicates the median, and the whiskers indicate the extreme values. Box‒Cox transformation parameters are equal to λ = 2 (**A**; *p*-value = 0.0047), λ = −0.05 (**B**; *p*-value < 0.0001), (*P*-values were estimated using Student’s t-test)

Finally, in order to validate our findings, we considered the uploaded gene expression dataset (GSE147472) for which the ECM3 status was also available as previously described [5], including 89 ECM3- and 42 ECM3 + HGBCs.We compared the distributions of SATB1 expression and PD-1 between ECM3- and ECM3 + patients and results show a reduced expression of PD1 expression, paralleled by increased SATB1 expressions in ECM3 + patients (Fig. 7).

## Discussion

This study showed that the ECM status in HGBC may influence adaptive T cell immunity by modulating regulatory T cell activity. In particular, the matricellular protein SPARC, a functional gene within the ECM3 signature, has been shown to enhance the suppressive activity of Tregs by repressing PD-1.

The immune checkpoint PD-1 is expressed by T cells upon activation and remains expressed even in the exhausted state. In healthy subjects, PD-1 triggering by the cognate ligand PD-L1 protects against autoimmune-related events, whereas in the tumor context, triggering PD-L1 is detrimental because it causes deactivation/inhibition of the T-cell response.

Other than effector T cells, PD-1 is also expressed by Tregs. By performing suppression assays using PD-1 + and PD-1^*neg*^ Tregs, we found that the latter were more suppressive than the PD-1 + counterpart, supporting the concept that PD-1 might restrain Treg activities and mark exhausted Tregs, as previously suggested [19]. Similarly, Kamada et al. reported that PD-1 deficiency, or PD-1 blockade, in Treg cells augments their proliferation and immunosuppressive activity in vitro and inhibits antitumor immunity in vivo [20]. These authors also proposed that hyperprogressive disease (HPD), a type of rapid tumor progression that occurs in some patients after immune checkpoint inhibitor treatment [21], could be due to blockade of the PD-1/PD-L1 axis in PD-1 + “exhausted” Tregs, which are present at a relatively high frequency in the microenvironment of tumors undergoing HPD [20]. More recently, Kumagai et al. reported that lactic acid induces PD-1 expression by Treg cells in highly glycolytic tumors and that PD-1 blockade invigorates PD-1-expressing Treg cells, resulting in treatment failure [22].

In this work, we demonstrated that the extracellular matrix, through the repression of PD-1, can by itself reinvigorate the activity of regulatory T cells, suggesting that for specific subtype of tumors (ECM3 +) an approach capable of interfering with Treg suppressive activity (e.g., anti-CTLA4, anti-OX40) could be more effective and less detrimental than inhibitors of the PD-1/PD-L1 axis, which might again reactivate Tregs. For this reason, a future approach could be to use the 8-gene qPCR-based classifier, as it would allow, at the time of biopsy, to identify patients who need a different immunotherapeutic strategy or therapeutic combinations. Given the relevance of immune checkpoint expression in both T cells and Tregs, we would like to highlight that, although our murine models suggest a coordinated regulation of PD-1, TIM-3, and LAG-3, corresponding data in human Tregs from ECM3-stratified tumors are currently lacking. Nonetheless, exploring immune checkpoint expression in human ECM3⁺ Tregs could offer valuable insights into their immunosuppressive function and responsiveness to targeted immunotherapies. Further studies will be crucial to elucidate these relationships in patients, ultimately enabling more precise and effective immunotherapeutic interventions.

As a proof of concept, we investigated whether IL-23 blockade could be a potential strategy to revert the immune suppressive microenvironment of ECM3 tumors. Similarly, Wertheimeier et al. recently reported that the IL-23/IL-23R axis maintains and stabilizes highly suppressive tumor-associated Tregs, suggesting that IL-23 blockade could be a possible immunotherapeutic strategy [23]. Our findings show that IL-23 inhibition effectively increases the population of Ki-67- PD-1 + Tregs, accompanied by a significant increase in CD8 + T-cell activation. Additionally, we demonstrated that anti-IL-23 treatment does not affect PD-1 expression on CD8 + T cells, likely because of the absence of the IL-23 receptor on these cells. However, this treatment alone was insufficient to produce measurable effects on tumor growth or metastasis. The limited overall infiltration of these tumors may contribute to the lack of observable phenotypic changes. Combining this approach with chemotherapy or with drugs that can convert a cold TME into a hot TME could be a promising strategy, and directly targeting SATB1 is also worth considering.

Indeed, the flavonoid baicalein has been shown to inhibit EMT and suppress metastasis by downregulating SATB1 and the Wnt/β-catenin pathway in BC [24]. Moreover, hydrophobic statins such as fluvastatin and simvastatin have been reported to downregulate SATB1 at the posttranslational level in a time- and dose-dependent manner in human colon cancer cells [25].

Another approach to activate the immune microenvironment of ECM3 BC is to directly target SPARC secretion at its source in tumor cells. SPARC secretion is linked to metabolic changes in the tumor microenvironment that promote immune suppression [26]. Moreover, in both melanoma and high-grade breast cancer cells, we demonstrated that reducing SPARC secretion, either through oleic acid administration or forced production of SCD5, has beneficial effects on antitumor immunity [13].

ECM3 + HGBC represents an aggressive disease with a limited response to therapy and is characterized by extensive matrix deposition, stiffness, and a unique immune microenvironment devoid of effector T lymphocytes and rich in myeloid cells. A thorough reprogramming of its microenvironment might provide better therapeutic options for ECM3 + HGBC patients. Targeting the secretion of SPARC even metabolically could be a successful approach.

## Conclusion

The extracellular matrix (ECM) plays a key role in immune regulation and therapy resistance. Here, we show that the ECM can repress PD-1 expression on regulatory T cells (Tregs) unleashing their suppressive activity and limiting CD8⁺ T-cell infiltration. In prospectively enrolled HGBC patients and gene profiles, we observed lower PD-1 expression in ECM3⁺ tumors, due to both Treg activation and impaired CD8⁺ recruitment. These findings highlight ECM3 as a potential biomarker of resistance to PD-1/PD-L1 immune checkpoint blockade (ICB), suggesting that ECM3⁺ patients may benefit from treatment with alternative checkpoint inhibitors beyond PD-1/PD-L1.

## Supplementary Information

Supplementary Material 1: Supplementary Table 1*. Panel of human antibodies used for flow cytometry.* The table lists the primary antibodies used for flow cytometry analysis of human PB, with details of the conjugated fluorophores, clones, manufacturing companies, category of reference numbers, and RRIDs provided by the Resource Identification Portal Community (https://rrid.site/data/source/nif-0000-07730-1/search). Supplementary Table 2. *Panel of mouse antibodies used for flow cytometry.* The table lists the primary antibodies used for flow cytometry analysis of mouse PB, with details of the conjugated fluorophores, clones, manufacturing companies, category of reference numbers, and RRIDs provided by the Resource Identification Portal Community (https://rrid.site/data/source/nif-0000-07730-1/search). 

Supplementary Material 2: Supplementary Fig. 1. Gating strategy to identify Treg populations in PB of HGBC patients. Lymphocytes were identified based on their forward- and side-scatter properties. CD4 T cells and CD8 T cells were identified and within CD4 gate we characterized Tregs that co-express CD25 and transcription factor forkhead box P3 (Foxp3). We further characterized Treg using Ki-67 and PD-1. Supplementary Fig. 2*. SPARC expression in different BC cell lines and the correlation between PD-1 on expression on Tregs with SPARC levels in the SN25/SN25ASP models.* A. Analysis of SPARC expression in four cell lines revealed that 4T1cl5 naturally exhibited high levels of SPARC, whereas the shRNA variant (4T1cl5sp548) was *SPARC*-silenced, as expected. The 4T1 cell line is the parental line, which expresses low levels of SPARC, whereas the recombinant 4T1SP counterpart specifically expresses the SPARC protein. B. MFI of PD-1 on Tregs from SN25A (*Sparc-deficient*) or SN25ASP (*Sparc-high*) tumors (*p*-value: 0.029). P-value was estimated using nonparametric Wilcoxon test. C. Semiquantitative qPCR analysis of *Satb1* and *Pd-1* (D) expression in FACS-sorted intratumor Tregs isolated from 4T1cl5 or 4T1cl5sp548 cells. Supplementary Fig. 3. *IL-23 expression in tumor cells and its correlation with SPARC levels.* A. qPCR analysis of *Il-23a* expression in 4T1 and 4T1SPARC cells (*p*-value: 0.258), 4T1cl5 and 41c15sp548 cells (B; p-value: 0.095), and SN25A (*SPARC*-KO) and SN25ASP (*SPARC*-high) BC cells (C; *p*-value: 0.001). P-values were estimated using nonparametric Wilcoxon test. D. IF analysis of SN25A and SN25ASP cells stained with Dapi, a marker of nuclear DNA, and antibodies against IL-23.

## Data Availability

The datasets generated during the current study are available in the GEO repository, with accession number GSE 282777 ([https://www.ncbi.nlm.nih.gov/geo/query/acc.cgi?acc=GSE282777](https://www.ncbi.nlm.nih.gov/geo/query/acc.cgi?acc=GSE282777)).

## Abbreviations

ACK: Ammonium chloride potassium
APC: Antigen presenting cell
BC: Breast cancer
BSA: Bovine serum albumin
ECM: Extracellular matrix
EMT: Epithelial to mesenchymal transition
FBS: Fetal bovine serum
HGBC: High-grade breast cancer
HPD: Hyperprogressive disease
HS: Heparan sulfate
IHC: Immunohistochemistry
IF: Immunofluorescence
LN: Lymph nodes
MEM: Minimum essential medium
MDSC: Myeloid-derived suppressor cell
MFI: Mean Fluorescence Intensity
PB: Peripheral blood
PBMC: Peripheral blood mononuclear cells
PD-1: Programmed cell death 1
qPCR: Quantitative polymerase chain reaction
RT: Room temperature
SPARC: Secreted Protein Acidic and Rich in Cysteine
SPL: Spleen
Teff: Effector T cell
TBS: Tris-buffered saline
TM: Tumor
TME: Tumor microenvironment
Treg: Regulatory T cell

## References

[1] Pickup MW, Mouw JK, Weaver VM. The extracellular matrix modulates the hallmarks of cancer. EMBO Rep. 2014;15(12):1243–53. 25381661 10.15252/embr.201439246 PMC4264927

[2] Giussani M, Landoni E, Merlino G, Turdo F, Veneroni S, Paolini B, et al. Extracellular matrix proteins as diagnostic markers of breast carcinoma. J Cell Physiol. 2018;233(8):6280–90. 29521413 10.1002/jcp.26513

[3] Robertson C. The extracellular matrix in breast cancer predicts prognosis through composition, splicing, and crosslinking. Exp Cell Res. 2016;343(1):73–81. 26597760 10.1016/j.yexcr.2015.11.009

[4] Bergamaschi A, Tagliabue E, Sorlie T, Naume B, Triulzi T, Orlandi R, et al. Extracellular matrix signature identifies breast cancer subgroups with different clinical outcome. J Pathol. 2008;214(3):357–67. 18044827 10.1002/path.2278

[5] Lecchi M, Verderio P, Cappelletti V, De Santis F, Paolini B, Monica M, et al. A combination of extracellular matrix- and interferon-associated signatures identifies high-grade breast cancers with poor prognosis. Mol Oncol. 2021;15(5):1345–57. 33523584 10.1002/1878-0261.12912 PMC8096783

[6] Sangaletti S, Tripodo C, Santangelo A, Castioni N, Portararo P, Gulino A, et al. Mesenchymal transition of high-grade breast carcinomas depends on extracellular matrix control of myeloid suppressor cell activity. Cell Rep. 2016;17(1):233–48. 27681434 10.1016/j.celrep.2016.08.075

[7] Horn LA, Chariou PL, Gameiro SR, Qin H, Iida M, Fousek K, et al. Remodeling the tumor microenvironment via blockade of LAIR-1 and TGF-beta signaling enables PD-L1-mediated tumor eradication. J Clin Invest. 2022;132(8):e155148. 35230974 10.1172/JCI155148 PMC9012291

[8] O’Connor RS, Hao X, Shen K, Bashour K, Akimova T, Hancock WW, et al. Substrate rigidity regulates human T cell activation and proliferation. J Immunol. 2012;189(3):1330–9. 22732590 10.4049/jimmunol.1102757 PMC3401283

[9] Martinez HA, Koliesnik I, Kaber G, Reid JK, Nagy N, Barlow G, et al. Regulatory t cells use heparanase to access IL-2 bound to extracellular matrix in inflamed tissue. Nat Commun. 2024;15(1): 1564. 38378682 10.1038/s41467-024-45012-9 PMC10879116

[10] Miller JA, Cai C, Langfelder P, Geschwind DH, Kurian SM, Salomon DR, et al. Strategies for aggregating gene expression data: the collapseRows R function. BMC Bioinformatics. 2011;12: 322. 21816037 10.1186/1471-2105-12-322 PMC3166942

[11] Savas P, Virassamy B, Ye C, Salim A, Mintoff CP, Caramia F, et al. Single-cell profiling of breast cancer T cells reveals a tissue-resident memory subset associated with improved prognosis. Nat Med. 2018;24(7):986–93. 29942092 10.1038/s41591-018-0078-7

[12] Osborne J. Improving your data transformations: applying the box-cox transformation. Pract Assess Res Eval. 2010;15(1):12. 10.7275/qbpc-gk17.

[13] Bellenghi M, Talarico G, Botti L, Puglisi R, Tabolacci C, Portararo P, et al. SCD5-dependent inhibition of SPARC secretion hampers metastatic spreading and favors host immunity in a TNBC murine model. Oncogene. 2022;41(34):4055–65. 35851846 10.1038/s41388-022-02401-y

[14] Cui H, Wang N, Li H, Bian Y, Wen W, Kong X, et al. The dynamic shifts of IL-10-producing Th17 and IL-17-producing Treg in health and disease: a crosstalk between ancient “Yin-Yang” theory and modern immunology. Cell Commun Signal. 2024;22(1): 99. 38317142 10.1186/s12964-024-01505-0 PMC10845554

[15] Wang B, Ji L, Bian Q. SATB1 regulates 3D genome architecture in T cells by constraining chromatin interactions surrounding CTCF-binding sites. Cell Rep. 2023;42(4): 112323. 37000624 10.1016/j.celrep.2023.112323

[16] Stephen TL, Payne KK, Chaurio RA, Allegrezza MJ, Zhu H, Perez-Sanz J, et al. SATB1 expression governs epigenetic repression of PD-1 in tumor-reactive T cells. Immunity. 2017;46(1):51–64. 28099864 10.1016/j.immuni.2016.12.015 PMC5336605

[17] Jawale D, Khandibharad S, Singh S. Decoding systems immunological model of sphingolipids with IL-6/IL-17/IL-23 axes in *L. major* infection. Biochimica et Biophysica Acta (BBA). 2023;1868(2): 159261. 10.1016/j.bbalip.2022.159261 36494028

[18] Pastor-Fernandez G, Mariblanca IR, Navarro MN. Decoding IL-23 signaling cascade for new therapeutic opportunities. Cells. 2020. 10.3390/cells9092044. 32906785 10.3390/cells9092044 PMC7563346

[19] Lowther DE, Goods BA, Lucca LE, Lerner BA, Raddassi K, van Dijk D, et al. PD-1 marks dysfunctional regulatory T cells in malignant gliomas. JCI Insight. 2016;1(5):e85935. 27182555 10.1172/jci.insight.85935 PMC4864991

[20] Kamada T, Togashi Y, Tay C, Ha D, Sasaki A, Nakamura Y, et al. PD-1(+) regulatory T cells amplified by PD-1 blockade promote hyperprogression of cancer. Proc Natl Acad Sci U S A. 2019;116(20):9999–10008. 31028147 10.1073/pnas.1822001116 PMC6525547

[21] Ferrara R, Mezquita L, Texier M, Lahmar J, Audigier-Valette C, Tessonnier L, et al. Comparison of fast-progression, hyperprogressive disease, and early deaths in advanced non-small-cell lung cancer treated with PD-1/PD-L1 inhibitors or chemotherapy. JCO Precis Oncol. 2020;4:829–40. 35050757 10.1200/PO.20.00021

[22] Kumagai S, Koyama S, Itahashi K, Tanegashima T, Lin YT, Togashi Y, et al. Lactic acid promotes PD-1 expression in regulatory T cells in highly glycolytic tumor microenvironments. Cancer Cell. 2022;40(2):201-18 e9. 35090594 10.1016/j.ccell.2022.01.001

[23] Wertheimer T, Zwicky P, Rindlisbacher L, Sparano C, Vermeer M, de Melo BMS, et al. IL-23 stabilizes an effector T(reg) cell program in the tumor microenvironment. Nat Immunol. 2024;25(3):512–24. 38356059 10.1038/s41590-024-01755-7 PMC10907296

[24] Ma X, Yan W, Dai Z, Gao X, Ma Y, Xu Q, et al. Baicalein suppresses metastasis of breast cancer cells by inhibiting EMT via downregulation of SATB1 and Wnt/beta-catenin pathway. Drug Des Devel Ther. 2016;10:1419–41. 27143851 10.2147/DDDT.S102541 PMC4841441

[25] Lakshminarayana Reddy CN, Vyjayanti VN, Notani D, Galande S, Kotamraju S. Down-regulation of the global regulator SATB1 by statins in COLO205 colon cancer cells. Mol Med Rep. 2010;3(5):857–61. 21472326 10.3892/mmr.2010.338

[26] Young CM, Beziaud L, Dessen P, Madurga Alonso A, Santamaria-Martinez A, Huelsken J. Metabolic dependencies of metastasis-initiating cells in female breast cancer. Nat Commun. 2023;14(1):7076.10.1038/s41467-023-42748-8 37925484 10.1038/s41467-023-42748-8 PMC10625534

