# Supplementary figures and images for: ECM-Induced IL-23 Drives Immune Suppression in Breast Cancer via Regulating PD-1 on Tregs
Source: J Exp Clin Cancer Res. 2025 Sep 1;44:264. doi: 10.1186/s13046-025-03518-0 (PMC12400771; doi:10.1186/s13046-025-03518-0)

## Slide 1
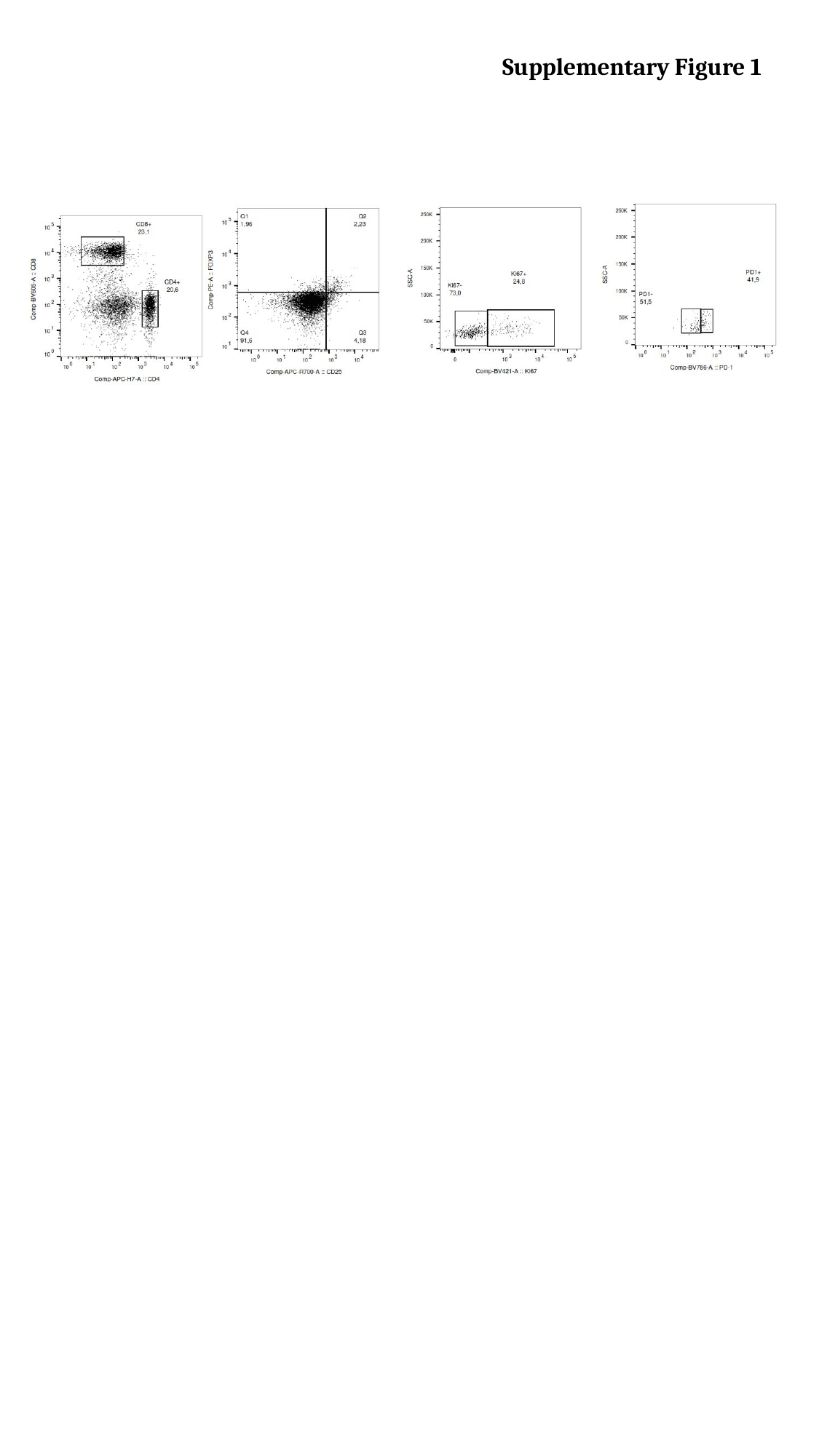

Supplementary Figure 1

## Slide 2
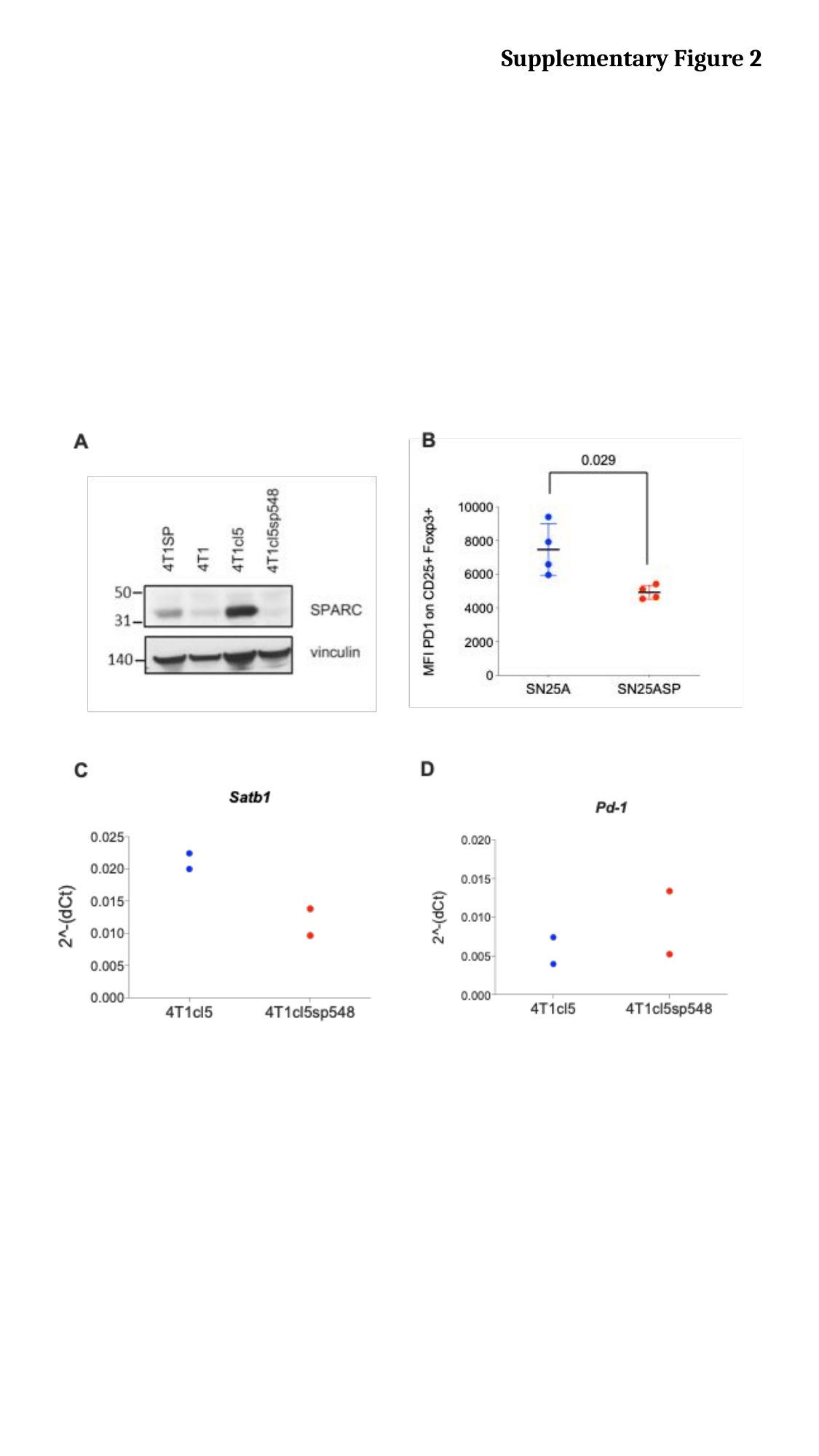

Supplementary Figure 2

## Slide 3
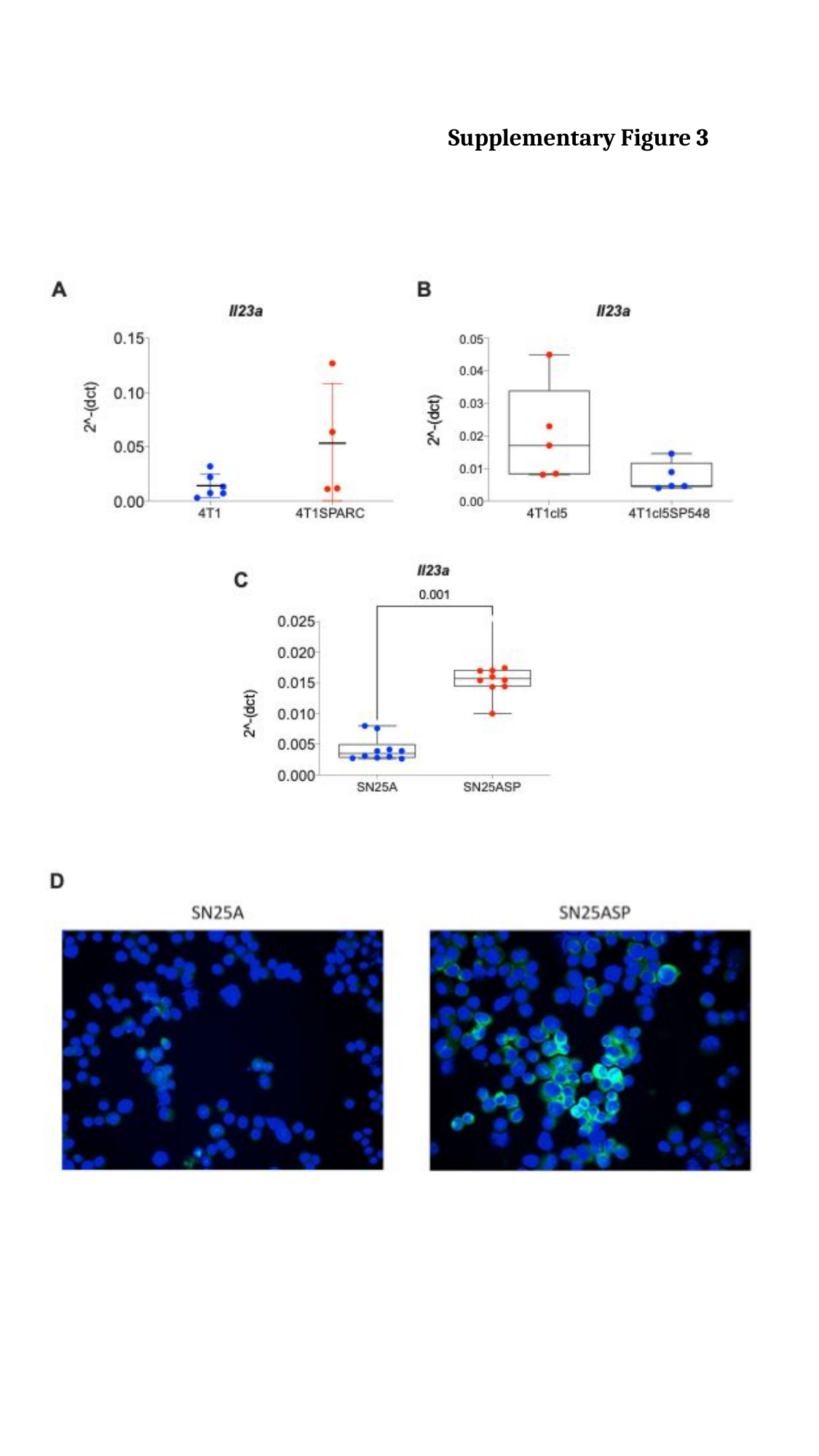

Supplementary Figure 3

Supplement: Supplementary file 1 — Supplementary Material 1: Supplementary Table 1. Panel of human antibodies used for flow cytometry. The table lists the primary antibodies used for flow cytometry analysis of human PB, with details of the conjugated fluorophores, clones, manufacturing companies, category of reference numbers, and RRIDs provided by the Resource Identification Portal Community (https://rrid.site/data/source/nif-0000-07730-1/search). Supplementary Table 2. Panel of mouse antibodies used for flow cytometry. The table lists the primary antibodies used for flow cytometry analysis of mouse PB, with details of the conjugated fluorophores, clones, manufacturing companies, category of reference numbers, and RRIDs provided by the Resource Identification Portal Community (https://rrid.site/data/source/nif-0000-07730-1/search). [file 13046_2025_3518_MOESM1_ESM.pptx]
