# Supplementary material for: ECM-Induced IL-23 Drives Immune Suppression in Breast Cancer via Regulating PD-1 on Tregs
Source: J Exp Clin Cancer Res. 2025 Sep 1;44:264. doi: 10.1186/s13046-025-03518-0 (PMC12400771; doi:10.1186/s13046-025-03518-0)
Supplement: Supplementary file 2 — Supplementary Material 2: Supplementary Fig. 1. Gating strategy to identify Treg populations in PB of HGBC patients. Lymphocytes were identified based on their forward- and side-scatter properties. CD4 T cells and CD8 T cells were identified and within CD4 gate we characterized Tregs that co-express CD25 and transcription factor forkhead box P3 (Foxp3). We further characterized Treg using Ki-67 and PD-1. Supplementary Fig. 2. SPARC expression in different BC cell lines and the correlation between PD-1 on expression on Tregs with SPARC levels in the SN25/SN25ASP models. A. Analysis of SPARC expression in four cell lines revealed that 4T1cl5 naturally exhibited high levels of SPARC, whereas the shRNA variant (4T1cl5sp548) was SPARC-silenced, as expected. The 4T1 cell line is the parental line, which expresses low levels of SPARC, whereas the recombinant 4T1SP counterpart specifically expresses the SPARC protein. B. MFI of PD-1 on Tregs from SN25A (Sparc-deficient) or SN25ASP (Sparc-high) tumors (p-value: 0.029). P-value was estimated using nonparametric Wilcoxon test. C. Semiquantitative qPCR analysis of Satb1 and Pd-1 (D) expression in FACS-sorted intratumor Tregs isolated from 4T1cl5 or 4T1cl5sp548 cells. Supplementary Fig. 3. IL-23 expression in tumor cells and its correlation with SPARC levels. A. qPCR analysis of Il-23a expression in 4T1 and 4T1SPARC cells (p-value: 0.258), 4T1cl5 and 41c15sp548 cells (B; p-value: 0.095), and SN25A (SPARC-KO) and SN25ASP (SPARC-high) BC cells (C; p-value: 0.001). P-values were estimated using nonparametric Wilcoxon test. D. IF analysis of SN25A and SN25ASP cells stained with Dapi, a marker of nuclear DNA, and antibodies against IL-23. [file 13046_2025_3518_MOESM2_ESM.docx]

**Supplementary Table 1**

**

**Supplementary Table 1*.*** *Panel of human antibodies used for flow cytometry.* The table lists the primary antibodies used for flow cytometry analysis of human PB, with details of the conjugated fluorophores, clones, manufacturing companies, category of reference numbers, and RRIDs provided by the Resource Identification Portal Community (https://rrid.site/data/source/nif-0000-07730-1/search).

**Supplementary Table 2**

**

**Supplementary Table 2.** *Panel of mouse antibodies used for flow cytometry.* The table lists the primary antibodies used for flow cytometry analysis of mouse PB, with details of the conjugated fluorophores, clones, manufacturing companies, category of reference numbers, and RRIDs provided by the Resource Identification Portal Community (https://rrid.site/data/source/nif-0000-07730-1/search).
